# Supplementary material for: A strong start for sustained success: inclusivity through a national group mentorship program for first-year graduate students
Source: bioRxiv. 2026 Mar 15:2026.03.12.710679. Preprint. [Version 1] doi: 10.64898/2026.03.12.710679 (PMC13061050; doi:10.64898/2026.03.12.710679)
Supplement: Supplement 1 — Supplementary File 1. Descriptions of each CL-GSEC team, their general function, and their size/management. [file media-1.pdf]

Supplementary File 1. Descriptions of each CL-GSEC team, their general function, and their size/management.

CL-GSEC Team	General Function	Description	Size/Management
Executive	Leadership	In charge of all internal operational aspects of CL-GSEC, including supporting each team in coordinating, organizing, and executing their roles and the overall team mission; as well representing CL-GSEC with the rest of Cientifico Latino, *Inc* and external organizations.	2 Co-Directors
Recruitment	Logistics/Preparation Support	Recruit, screen, and match mentors and mentees in spring/summer for CL-GSEC program in academic year	Lead + 2 team members
Mentor-Mentee Relations	Mentee Resources	Main contact and manager of CL-GSEC mentorship small groups; liaison for conflict resolution	Lead + 2 team members
GradSchool 101 Workshops	Mentee Resources	Plan and host a series of panels covering general topics relevant for Grad School's first year students.	Lead + 2 team members
Community Engagement	Mentee Resources	Create a sense of community in the CL-GSEC cohort. Plan, execute, and host virtual social events (3x/year). Develop new community engagement initiatives (e.g., in person meet-ups).	Lead + 2 team members
Marketing	Operations Support	Advertise CL-GSEC events & announcements (including generating ads & social media)	Lead + 2 team members
Data	Operations Support	Maintain, organize, and analyze all the data gathered from CL-GSEC's operations. (application, pre-, post-, and check-in surveys)	Lead + 2 team members
